# *POLG2*-Linked Mitochondrial Disease: Functional Insights from New Mutation Carriers and Review of the Literature

**DOI:** 10.1007/s12311-023-01557-x

**Published:** 2023-04-22

**Authors:** Max Borsche, Marija Dulovic-Mahlow, Hauke Baumann, Sinem Tunc, Theresa Lüth, Susen Schaake, Selin Özcakir, Ana Westenberger, Alexander Münchau, Evelyn Knappe, Joanne Trinh, Norbert Brüggemann, Katja Lohmann

**Affiliations:** 1 Institute of Neurogenetics, University of Lübeck, Lübeck, Germany; 2 Department of Neurology, University of Lübeck and University Hospital Schleswig-Holstein, Campus Lübeck, Lübeck, Germany; 3 Institute of Systems Motor Science, University of Lübeck, Lübeck, Germany

**Keywords:** POLG2, Adult-onset ataxia, Mitochondrial dysfunction

## Abstract

Different pathogenic variants in the *DNA polymerase-gamma2 (POLG2)* gene cause a rare, clinically heterogeneous mitochondrial disease. We detected a novel *POLG2* variant (c.1270 T > C, p.Ser424Pro) in a family with adult-onset cerebellar ataxia and progressive ophthalmoplegia. We demonstrated altered mitochondrial integrity in patients’ fibroblast cultures but no changes of the mitochondrial DNA were found when compared to controls. We consider this novel, segregating *POLG2* variant as disease-causing in this family. Moreover, we systematically screened the literature for *POLG2*-linked phenotypes and re-evaluated all mutations published to date for pathogenicity according to current knowledge. Thereby, we identified twelve published, likely disease-causing variants in 19 patients only. The core features included progressive ophthalmoplegia and cerebellar ataxia; parkinsonism, neuropathy, cognitive decline, and seizures were also repeatedly found in adult-onset heterozygous *POLG2*-related disease. A severe phenotype relates to biallelic pathogenic variants in *POLG2, *i.e., newborn-onset liver failure, referred to as mitochondrial depletion syndrome. Our work underlines the broad clinical spectrum of *POLG2*-related disease and highlights the importance of functional characterization of variants of uncertain significance to enable meaningful genetic counseling.

**Supplementary Information:**

The online version contains supplementary material available at 10.1007/s12311-023-01557-x.

## Introduction

Mitochondria are important cell organelles fulfilling essential tasks in cells, including energy production. They have their own genetic material (mitochondrial DNA, mtDNA) encoding components of the respiratory chain. Mutations in mtDNA, either inherited or acquired, result in diverse clinical phenotypes [1]. mtDNA is replicated by DNA polymerase gamma, which is composed of a catalytic (encoded by *POLG*) and an accessory subunit (encoded by *POLG2*) [2]. Mutations in both nuclear genes lead to variable expression of inherited movement and multisystem disorders associated with mitochondrial dysfunction. While *POLG* mutations are rather common and have been reviewed in 2019 [3], available information about *POLG2* mutations is scarce. To date, the few reported heterozygous *POLG2* mutations showed a disease onset in adulthood and were linked to (chronic) progressive external ophthalmoplegia ((c)PEO) and cerebellar ataxia leading to the syndrome of “progressive external ophthalmoplegia with mtDNA deletions, autosomal dominant ataxia type 4” (PEOA4, OMIM #610,131) [4]. Furthermore, biallelic mutations in *POLG2* cause a severe infancy-onset phenotype referred to as “mitochondrial DNA depletion syndrome” (OMIM #618,528 or #619,425), leading to liver failure and death in infancy [5]. Here, we describe clinical features and the functional characterization of two siblings with a novel, heterozygous likely pathogenic *POLG2* missense variant (c.1270 T > C; p.Ser424Pro). We further compare the phenotype to other mutation carriers by reviewing all *POLG2* mutations, including the re-evaluation of the pathogenicity of variants from the published literature.

## Methods

### Patients

The study was approved by the local ethics committee of the University of Lübeck and all participants gave written informed consent. Blood samples from three affected family members (including the index patient [L-10343], her brother [L-11048], and their mother [L-10914]) were genetically tested by exome sequencing. The index patient and her brother underwent a detailed clinical examination and skin biopsies were taken to establish primary skin fibroblast lines. No biopsy could be taken from the meanwhile deceased mother.

### Genetic Testing

After exclusion of several spinocerebellar ataxias due to repeat expansions, exome sequencing was performed at CENTOGENE GmbH (Rostock, Germany) in a diagnostic setting. The candidate variant in *POLG2* was confirmed by Sanger sequencing in all three affected family members.

### Functional Analysis

#### Cell Culture

Two healthy individuals (L-2152, L-2153) without pathogenic *POLG2* variants were included as controls in the functional characterization. Dermal skin fibroblast cultures were maintained in Dulbecco’s modified Eagle’s medium (DMEM, Life technologies) supplemented with 10% fetal bovine serum (Life technologies) and 1% penicillin/streptomycin (Life technologies) at 37 °C with 5% CO_2_.

### Mitochondrial DNA and Replication Analyses

For deep mitochondrial sequencing, we used blood samples from three patients and DNA from fibroblast cultures of both available patients. We also included four age- and sex-matched controls for each patient and DNA source. mtDNA was amplified by two overlapping long-range PCRs (FampA: 5′-AAATCTTACCCCGCCTGTTT-3′, FampB: 5′-GCCATACTAGTCTTTGCCGC-3′, RampA: 5′-AATTAGGCTGTGGGTGGTT-3′, RampB: 5′-GGCAGGTCAATTTCACTGGT-3′ [6]). Sequence analysis was performed at > 1000 × coverage on a GridION machine (Oxford Nanopore, Oxford, UK) as described [7], using Mutserve2 (v2.0.0) for mtDNA variant calling [8]. Furthermore, a real‐time PCR assay for detection of three targets within the mitochondrial genome (ND1 [NADH dehydrogenase 1], ND4, and D‐loop) was performed using blood samples from the three *POLG2* mutation carriers and three healthy controls as described [9, 10]. For mtDNA copy number quantification, the ND1 concentration per unit area was calculated. To detect mtDNA major arc deletions, the ND1:ND4 ratio was determined. During transcription and replication, a linear DNA molecule, that is, 7S DNA, is incorporated in this particular region of the mitochondrial genome, resulting in a triple‐stranded structure denominated as “D‐loop.” The D‐loop:ND1 ratio is therefore representative of the mtDNA replication status in a given cell, with a higher ratio representing more actively transcribed or replicating mtDNA molecules. All measurements were performed three times in duplicates. We also used long-range PCR of the mtDNA to test for the presence of shorter fragments due to partial mtDNA deletions.

### Immunofluorescence Staining and Image Analysis

For immunocytochemical analysis, cells on coverslips were fixed in 4% formaldehyde for 15 min, permeabilized, and blocked with 0.1% Triton X-100 in 4% normal goat serum in PBS for 1 h. Mitochondrial network interconnectivity was analyzed in cells stained for mitochondrial GRP75. Immunofluorescence staining was performed with primary antibody against GRP75 (1:1000; Abcam) and respective secondary fluorescence antibody (1:400; Life technologies). The images were taken as z-stacks using a confocal microscope. The form factor was calculated using the formula [*P*_m_^2^]/[4*πA*_m_], with *P*_m_ being the length of the mitochondrial outline and *A*_m_ being the area of the mitochondrion. At least ten cells from two coverslips per individual were analyzed and a mean form factor was calculated as a measure for mitochondrial interconnectivity using ImageJ (NIH software) as previously described [11].

### Western Blot Analysis

Cell pellets were extracted using SDS extraction buffer (50 mM Tris–HCl pH 7.6, 150 mM NaCl, 1% DOC, 1% NP-40, and 0.1% SDS) or 1% Triton X-100 lysis buffer (containing 10% glycerol, 150 mM NaCl, 25 mM Hepes pH7.4, 1 mM EDTA, 1.5 mM MgCl2, proteinase inhibitor cocktail) and gels were blotted onto nitrocellulose membranes. Antibodies used for immunoblotting were anti-TOMM20 (translocase of the outer mitochondrial membrane 20; 1:1,000; Santa Cruz) and anti-β-actin (1:1,000,000; Sigma).

### Mitochondrial Membrane Potential (MMP) Analysis

Fluorescence-activated cell sorting (FACS) was used to assess MMP. Primary skin fibroblasts were incubated with tetramethylrhodamine methyl ester perchlorate (TMRM, 10 nM). Subsequently, samples were washed to remove unbound dye. Carbonyl cyanide-p-trifluorometoxyphenylhydrazone (FCCP, 10 µM) was added to a part of the cells to suppress MMP. The TMRM-staining was measured using a BD LSR II® flow cytometer. First, living cells (4′,6-diamidino-2-phenylindole (DAPI)negative) were identified and used for the gating strategy. Then, the mean fluorescence intensity (MFI) of TMRM-stained cells was assessed in FCCP-treated fibroblasts compared to fibroblasts just diluted in medium. Results were calculated using the following formula: MFI TMRM^medium^ – MFI TMRM^FCCP^ = MMP. Data were analyzed with FlowJo® Software (Treestar).

### Statistical Analysis

Differences were analyzed using unpaired *t*-tests or analyses of variance (ANOVA) with a Bonferroni-Dunn post hoc test. The error bars indicate SEM of *n* ≥ 3 independent experiments. Age at onset, age at examination, and disease duration in the review part are shown as mean ± standard deviation. Statistical analyses were performed with GraphPad Prism 9.

### Review of the Literature

**Fig. 1:**
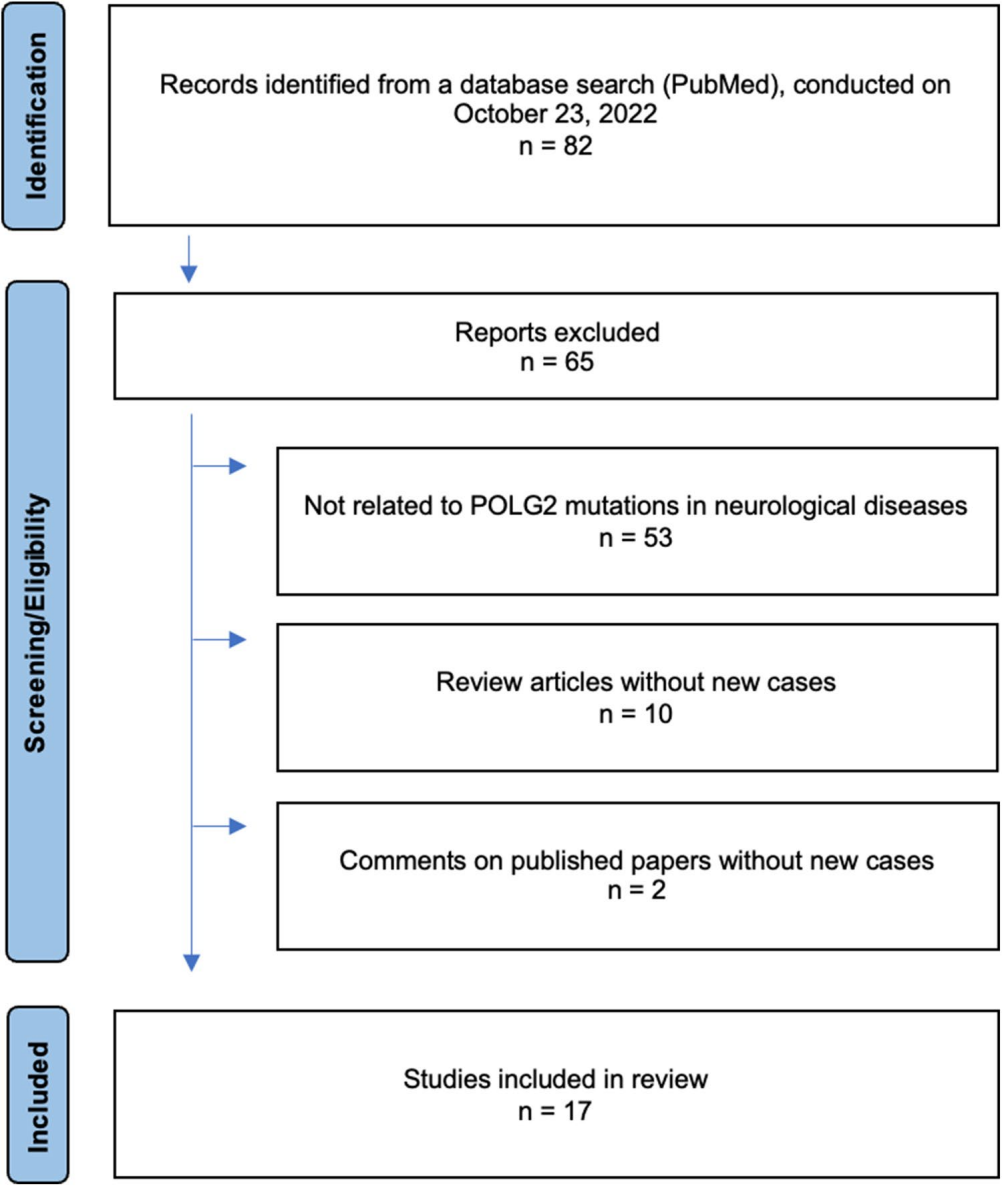
Flow chart of the search strategy of the literature review. The flow diagram according to the PRISMA guidelines presents the steps of literature research, the respective numbers of papers as well as reasons of exclusion

To identify the clinical signs and symptoms associated with *POLG2* mutations, we conducted a literature review in PubMed with the search term “POLG2” on October 23, 2022, identifying 82 publications (Fig. 1). We extracted clinical as well as genetic information systematically from all publications. For all identified *POLG2* variants, the frequency was analyzed using the GnomAD database (https://gnomad.broadinstitute.org/), the CADD (Combined Annotation Dependent Depletion) score [12] (https://cadd.gs.washington.edu/) was calculated, and a pathogenicity scoring was performed according to the MDSGene workflow as described (https://www.mdsgene.org/methods). Moreover, all variants were rated according to the ACMG (American College of Medical Genetics and Genomics) guidelines [13] using Franklin by Genoox (https://franklin.genoox.com/clinical-db/home).

Individuals harboring variants with a MAF > 0.01 at least in a certain population and/or variants rated as benign according to the ACMG criteria or by MDSGene scoring were disregarded as well as individuals with more than one *POLG2* variant on the same allele or additional variants in genes linked to similar phenotypes (Table S1).

## Results

### Case Reports

**Fig. 2:**
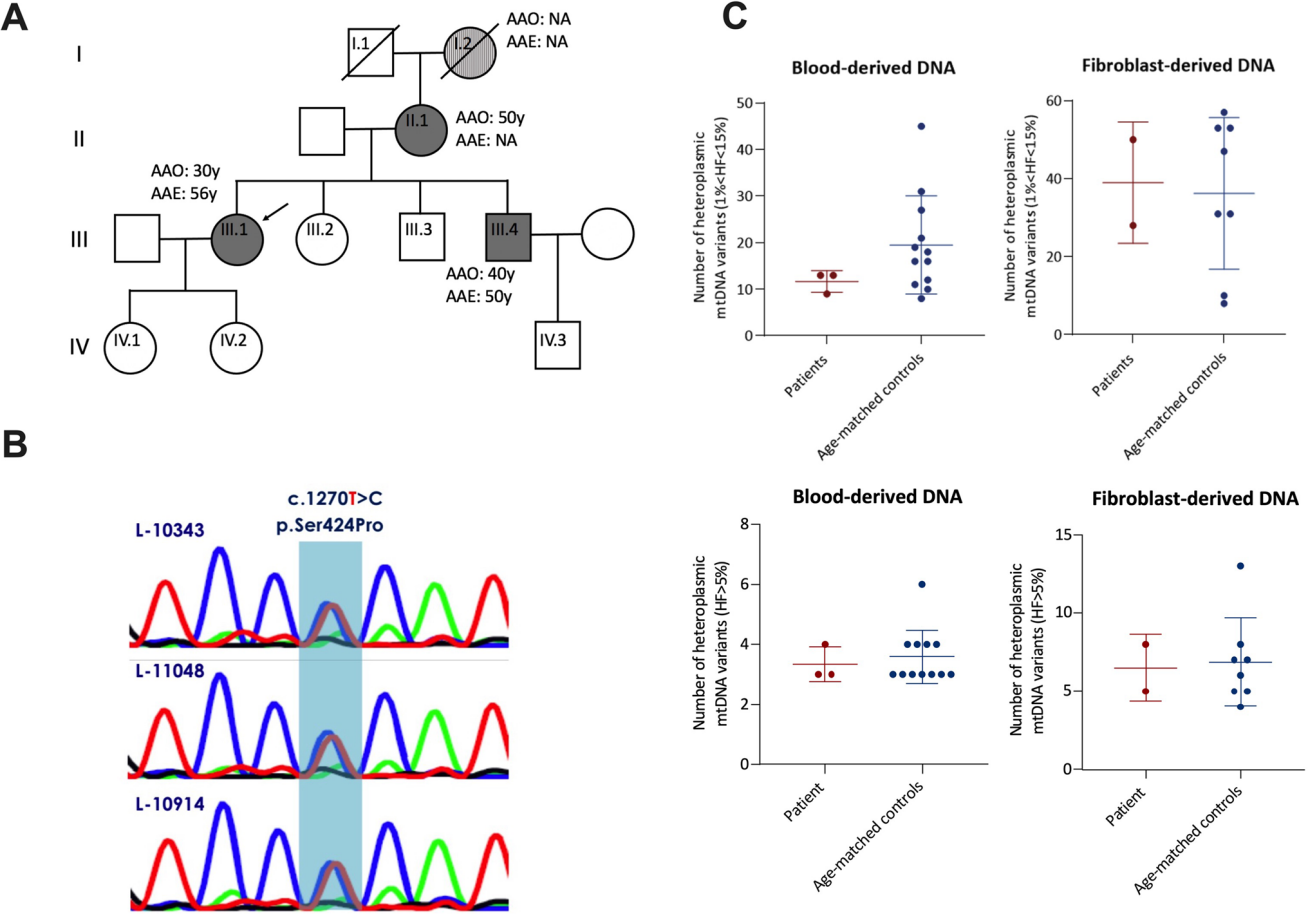
A novel heterozygous variant in *POLG2*. **A** Pedigree of the presented family harboring a heterozygous *POLG2* variant. Squares represent male individuals; circles represent female family members. The index patient is marked with an arrow. Dark gray squares represent clinically affected family members with genetic confirmation of the c.1270 T > C (p.Ser424Pro) variant. The striped circle indicates that the index patient’s grandmother was reported to be affected. However, no clinical examination and no genetic testing were performed. Age at onset (AAO) and age at examination (AAE) are stated for affected family members if available (NA: not available). **B** Genomic DNA sequences of the c.1270 T > C variant are shown from blood. The traces are representative of 2 sequencing runs. **C** Mitochondrial DNA (mtDNA) sequence variants in *POLG2* mutation carriers. MtDNA was extracted from blood or cultured fibroblasts. The number of low-frequency heteroplasmic variants (1–15% heteroplasmy frequency [HF]) (upper panels) and higher frequency variants (HF > 5%) (lower panels). Each dot represents one sample, and median and 1st and 3rd quartile are indicated

We report two siblings with a heterozygous *POLG2* variant exhibiting cerebellar ataxia and progressive ophthalmoplegia in one sibling and dysmetria of saccades in the other sibling. The index patient L-10343 (Fig. 2A, Video 1), a 56-year-old female patient of German origin, initially experienced gait imbalance at the age of 30 years. The disease has been slowly progressive: 10 years after disease onset, the patient developed diplopia. At the age of 50 years, diplopia was so pronounced that the patient tended to keep one eye closed to avoid disturbing visual impressions. Moreover, speech and gait also deteriorated. Cognitive problems, as well as autonomic symptoms, were denied. The neurological examination revealed bilateral ptosis and external ophthalmoplegia, manifesting by reduced eye bulb adduction more strongly pronounced on the right side. The patient had slowed and hypometric horizontal saccades whereas vertical saccades were normal. She exhibited dysarthria and severe cerebellar ataxia, including dysmetria during finger-chase and finger-nose testing, and an abnormal heel-shin slide, the latter more pronounced on the left side. She was unable to walk and to stand unaided requiring a wheelchair. Moreover, there was dystonic posturing of the hands and mild cervical dystonia with torticollis and laterocollis to the right side. She scored 27/40 points on the Scale for the assessment and rating of ataxia (SARA). Cognitive function was not impaired as revealed by 28/30 points on the Montreal cognitive assessment (MoCA). The patient had neither spasticity nor signs of neuropathy or parkinsonism. A cranial MRI was unremarkable.

Her youngest brother L-11048 (Fig. 2A, Video 2), a construction worker, developed balance problems at the age of 40 years, first manifesting while working on scaffolds. In the following years, he developed gait impairment, slightly slurred speech, and problems with fine motor tasks. The neurological examination at the age of 50 years revealed hypermetric saccades and macro square wave jerks but no ophthalmoplegia. Moreover, he had dysarthria, dysdiadochokinesia, hypermetric finger chase, and a slightly abnormal finger-nose test (SARA 10/40). His gait was broad-based and ataxic. Signs of neuropathy were present clinically (reduced sense of vibration) and in nerve conduction studies (mild sensory axonal neuropathy).

In both siblings, standard laboratory analysis, including creatinine kinase, liver enzymes (alanine aminotransferase and aspartate aminotransferase), and bilirubin, were unremarkable, as well as electroencephalography.

Another older sister and one younger brother were reported to be unaffected whereas the patients’ mother presented features of cerebellar ataxia commencing at the age of 50 years. Of note, also her maternal mother was reported to have suffered from ataxia collectively indicating an autosomal dominant or maternal mode of inheritance (Fig. 2A).

Spinocerebellar ataxias due to repeat expansions and pathogenic variants in 188 ataxia genes, including *POLG* but not *POLG2*, were excluded prior to exome sequencing in the index patient and her affected brother. By filtering the exome data for rare (minor allele frequency (MAF) < 0.01), heterozygous variants shared by the three affected individuals in genes previously linked to a mitochondrial phenotype, we detected a *POLG2* missense variant (NM_007215.3: c.1270 T > C; p.Ser424Pro) as the only candidate. This variant was confirmed by Sanger sequencing (Fig. 2B) and has not yet been reported in any patient but is adjacent to the previously reported p.Ser423Tyr variant [14]. It has also not been reported in gnomAD (~ 140,000 samples; https://gnomad.broadinstitute.org/gene/ENSG00000256525?dataset=gnomad_r2_1). The CADD score was 24.8 pointing to deleteriousness. Due to the rarity of the variant and the segregation in the family but in the absence of any published functional data, the variant was classified initially as variant of uncertain significance (VUS, ACMG criteria: PM2 PP1, PP3, PP4 [13]).

### Functional Analysis

To evaluate pathogenicity in vitro, we performed functional analyses to test for mitochondrial integrity in the *POLG2* variant carriers. We first looked for changes of the mtDNA. Notably, deep mtDNA sequencing did not reveal any differences when compared to controls (Fig. 2C), neither for low-frequency (somatic) heteroplasmic nor for high-frequency variants. Furthermore, we did also not identify large mtDNA deletions neither by quantitative PCR nor by long-range PCR (data not shown). Likewise, we did not observe changes in mtDNA copy number and replication status in the *POLG2* mutation carriers compared to healthy controls (data not shown).

**Fig. 3:**
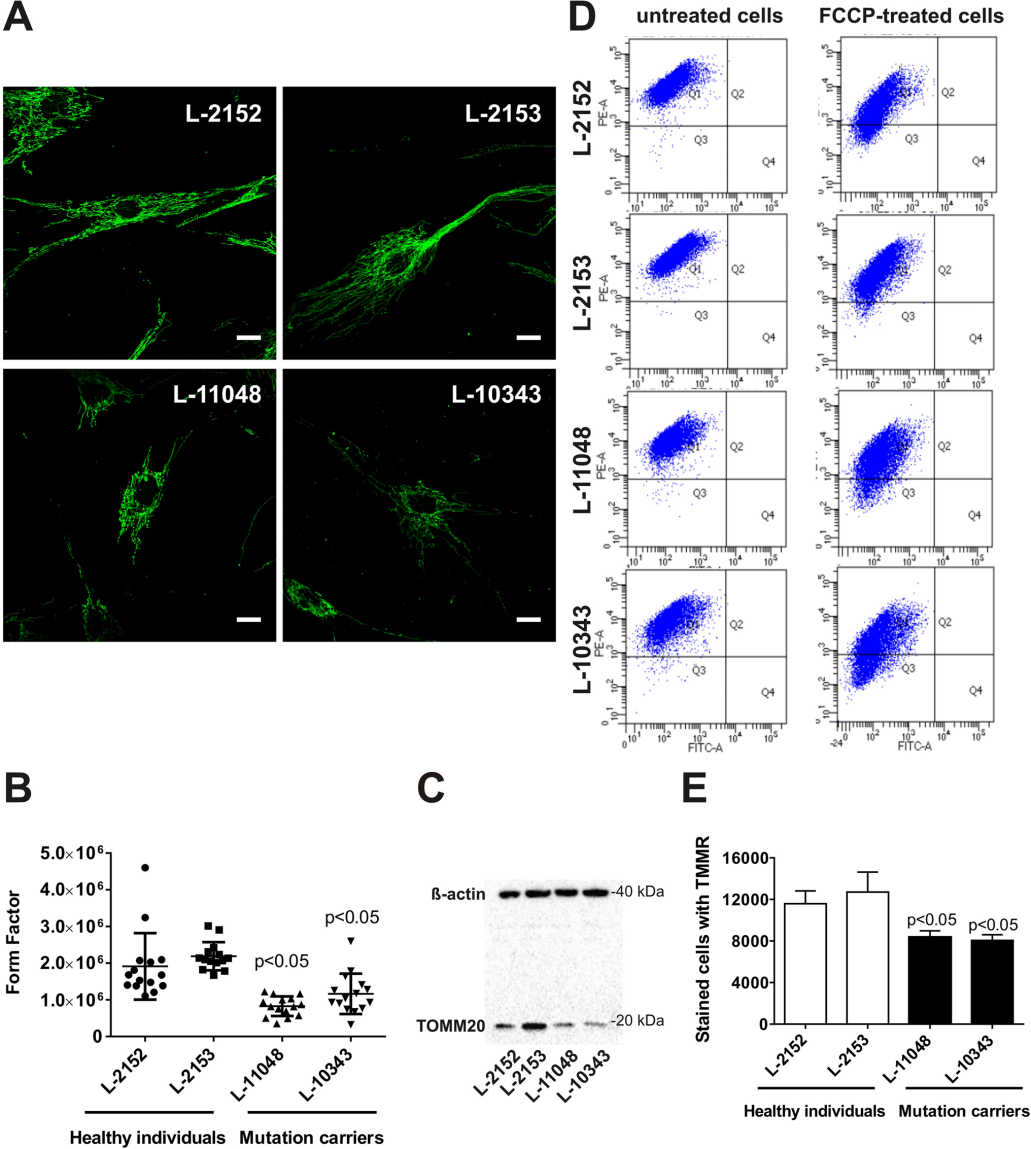
Altered mitochondrial network, decreased TOMM20 levels, and decreased mitochondrial membrane potential (MMP) in *POLG2*-mutant fibroblasts. *POLG2*-mutant fibroblasts from the index patient (L-10343), and her brother (L-11048), and two healthy control fibroblasts (L-2152, L-2153) were examined. **A** The mitochondrial network is shown using confocal microscopy in cells immunostained with anti-GRP75. The scales correspond 20 µm. **B** Form factor as a measure for mitochondrial network interconnectivity (GRP75 immunostaining) was calculated for the controls and the *POLG2-*mutants. Each dot represents the measurement in a single cell (*n* = 14). The mean values and the standard deviations of the investigated individuals are shown (*p* < 0.05, comparing *POLG2*-mutant fibroblasts with healthy controls). **C** Western blot analysis shows decreased mitochondrial TOMM20 protein levels in *POLG2*-mutant fibroblasts in comparison to controls with β-actin as loading control. **D** The number of tetramethylrhodamin-methylester (TMRM; 10 nM)-stained cells was assessed by flow cytometry under basal condition (left panel) or upon treatment with a mitochondrial uncoupler (depolarizer) carbonyl cyanide 4-(trifluoromethoxy)phenylhydrazone (FCCP; 10 µM) (right panel). Representative dot plots from two *POLG2-*mutant fibroblasts (L-11048, L-10343) and two healthy controls (L-2152, L-2153) are depicted. Each dot represents the fluorescence measurement in a single cell (*n* = 10,000). **E** Quantification of the TMRM-positive cells normalized to FCCP-treated cells by using FlowJo. The analysis revealed reduced number of cells stained with the TMRM dye, indicating decreased MMP in *POLG2-*mutant fibroblasts. The mean values and the standard deviations of the investigated individuals are shown (*n* = 3 experiments, *p* < 0.05, comparing MMP in *POLG2*-mutant fibroblasts with MPP in control fibroblasts)

Next, we analyzed the effect of mutant POLG2 on the integrity of the mitochondrial network by calculating the form factor in cultured *POLG2*-mutant fibroblasts and controls. The analysis revealed decreased mitochondrial branching and interconnectivity in *POLG2*-mutant fibroblasts from the index patient and her brother (Fig. 3A, B) when compared to controls (*p* < 0.05). In addition, we analyzed protein levels of the mitochondrial translocase TOMM20, located in the outer membrane, by Western blotting. TOMM20 protein levels were decreased in *POLG2*-mutant fibroblasts from both available patients compared to healthy controls (Fig. 3C). Furthermore, *POLG2*-mutant fibroblasts exhibited a decreased mitochondrial membrane potential (*p* < 0.05), analyzed by flow cytometry, compared to healthy controls (Fig. 3D, E). This suggests the variant c.1270 T > C (p.Ser424Pro) in *POLG2* as the cause of the complex neurological phenotype due to mitochondrial dysfunction in this family despite the absence of any detectable changes on mtDNA derived from blood and fibroblasts. As the diagnosis was made by genetic testing, a muscular biopsy was not performed.

Due to the detected functional changes in the mitochondrial integrity, a strong criterion from the ACMG recommendations was now fulfilled, we classified the variant as likely pathogenic (ACMG criteria: PS3, PM2 PP1, PP3, PP4 [13]).

### Review of the Literature

The literature search revealed only 17 reports containing clinical information for individual monoallelic (heterozygous) or biallelic (homozygous) *POLG2* variant carriers. After excluding patients with multiple variants on the same allele in *POLG2* or other genes linked to a similar phenotype [15–19], 24 patients with suggested *POLG2*-related disease carrying 14 different variants in the heterozygous state [4, 14, 20–25] remained. Five of these 14 variants (39%) did not fulfill the criteria of pathogenicity by MDSGene and/or scored “benign” or “likely benign” according to the ACMG guidelines and were excluded (Table S1). Out of the five excluded variants, three had a MAF > 0.01, at least in certain populations. The rating of variants according to state-of-the-art criteria of pathogenicity led to the exclusion of eight patients (Table S2). The remaining nine *POLG2* variants scored at least as possibly pathogenic and comprised three missense changes, three nonsense, one frameshift, one splice site variant, and one in-frame insertion (Table S2). Furthermore, all three homozygous variants reported in independent patients with recessive *POLG2*-related disease were scored at least possibly pathogenic according to the MDSGene and VUS/likely pathogenic according to ACMG criteria, respectively (Table S3).

Five publications focused on twelve adult-onset patients, while one paper reported on a patient examined at the age of 26 years that had exercise intolerance and myoclonus as core symptoms but was excluded from further analysis due to missing information on the age of onset [5, 17, 20–22] (Table S2). Among the remaining adult-onset cases (*n* = 12, 10 females), the age at onset was 52.2 ± 9.5 years, age at examination 66.2 ± 9.5 years, and disease duration 13.9 ± 5.9 years, respectively. Cerebellar ataxia was the most common feature reported in nine individuals (100% of cases with information on this item (*n* = 9)), followed by neuropathy in six of seven (86%), and PEO in eight of twelve (67%) patients. Ptosis was reported in three of eight (38%) and seizures were observed in three patients, while neither gastric nor liver problems were reported in any of the patients with onset in adulthood. Moreover, *POLG2* mutations manifested with parkinsonism in three individuals from the same Belgian pedigree [22] and one additional independent female patient [25], and “extrapyramidal signs” were reported in two, and “pyramidal signs” in four individuals from a recently published family [23] (Table 1, Table S2). In one case, camptocormia was the core symptom [24] (Table S2). Regarding muscle biopsy results, all six individuals with data on cytochrome C oxidase (COX) staining had COX-negative fibers and mtDNA deletions in muscle tissue were observed in all six patients in whom this feature was investigated (Table 1).

**Table 1:** Clinical features of the two individuals described in this study compared to adult-onset cases with heterozygous *POLG2* mutations from the literature

	L-10343	L-11048	Review of patients with adult-onset disease due to heterozygous *POLG2* mutations reported to date (*n* = 12)		
			Present	Percentage (of patients with information)	Percentage (of all patients)
PEO	+	−	8/12	67%	67%
Ptosis	+	−	3/8	38%	25%
Cerebellar ataxia	+	+	9/9	100%	75%
Exercise intolerance	−	−	3/3	100%	25%
Neuropathy	−	+	6/7	86%	50%
Parkinsonism	−	−	4/6	67%	33%
Cognitive impairment	−	nt	5/7	71%	42%
Seizures	−	−	3/5	60%	25%

Only individuals with onset in adulthood (age at onset > 18y) were included. *n*, number of individuals; *PEO*, progressive external ophthalmoplegia; + , present; − , absent; *nt*, not tested. As information was not available for all items in all individuals, frequency is reported in percent for the individuals with information only as well as related to all individuals

One single screening study investigating 112 *POLG*-negative unrelated individuals with possible early-onset mitochondrial disease identified eleven childhood-onset cases with heterozygous *POLG2* mutations [14], of which three individuals (age at examination 5.7 ± 4.0 years, 1 female) had variants scored at least possibly pathogenic according to the criteria applied in our study. Regarding the phenotype, muscle weakness/hypotonia and liver disease were present in two cases, accompanied by gastric complaints and developmental delay in one individual.

The literature review also revealed three patients with homozygous *POLG2* missense variants. The first patient was a 3-month-old boy carrying the homozygous missense variant c.544C > T (p.Arg182Trp). He suffered from a mitochondrial DNA depletion syndrome manifesting as a fulminant liver failure with consecutive death [6, 26]. Other biallelic *POLG2* mutations in two independent individuals have been linked to epilepsy [27] and a combination of childhood-onset optic atrophy, cerebellar ataxia, peripheral neuropathy, psychiatric comorbidities, and premature ovarian failure [28], respectively (Table S3).

## Discussion

Here, we report three affected family members from an autosomal-dominant ataxia pedigree all harboring a novel likely pathogenic heterozygous *POLG2* variant. Two of these patients underwent extensive clinical examination. Their clinical presentation was in line with the previously observed phenotype of *POLG2*-related disease, including PEO in the index patient and cerebellar ataxia in both siblings.

Since the missense variant was of uncertain pathogenicity, we tested the mitochondrial integrity in blood and patient-derived cultured fibroblasts. We demonstrated that the mitochondrial network was altered, and TOMM20 protein levels as well as MMP were decreased in *POLG2-*mutant fibroblasts. The use of fibroblast cultures to model *POLG2*-related disease is supported by previous reports showing a reduction of MMP in the mutant [20, 23]. Notably, we did not observe changes in mtDNA, neither an increased number of low- or high-frequency single nucleotide variants when compared to age- and sex-matched controls, nor copy number changes or mtDNA deletions in our patients. The latter might be restricted to muscle cells. Interestingly, it was previously shown that even after ethidium bromide-induced mtDNA depletion, the restoration of mtDNA levels was not delayed in fibroblasts from *POLG2* patients [29]. This suggests that cells compensate for the mtDNA replication defect by slowing down mtDNA degradation, possibly explaining the lack of detectable changes on the mtDNA in blood and fibroblasts. Based on the segregation of the variant within the family, the characteristic phenotype, and the altered mitochondrial integrity, our data suggest that this variant is likely pathogenic and causes the mitochondrial phenotype in this family. Of note, functional analyses, as performed in the context of the novel variant presented here, are needed to classify newly identified variants for which pathogenicity is uncertain.

A review of the literature revealed that in contrast to *POLG*-linked disorders, *POLG2*-related disease is very rare — only 16 cases with heterozygous and three individuals with biallelic likely disease-causing *POLG2* variants have been published to date. Of note, more than a third of the variants described as causative for dominantly inherited *POLG2*-related disease in the literature did not meet the state-of-the-art criteria for pathogenicity scoring, stressing the urgent need to re-evaluate the impact of reported variants as soon as further data and new scoring recommendations become available. Importantly, the classification of variants according to the MDSGene criteria was consistent with the ACMG guidelines.

Similar to *POLG*-related disease, the clinical spectrum of heterozygous *POLG2* mutations comprises cerebellar ataxia and PEO in adulthood-onset and metabolic abnormalities and seizures in childhood-onset cases. Moreover, seizures may also be present in adult-onset patients, as well as parkinsonism and camptocormia. As expected, biallelic *POLG2* variants can lead to a more severe phenotype with early-onset liver failure and death, referred to as mtDNA depletion syndrome. Notably, cases with a later onset and a less severe phenotype with biallelic *POLG2* mutations have been published as well. MtDNA deletions and COX-negative fibers in muscle tissue have been reported in a few tested patients with *POLG2*-related disease due to heterozygous mutations. However, COX-negative fibers, as well as the clinical symptoms, have a significant overlap with the clinical picture of *POLG*-related disease and other mitochondrial conditions, which appears plausible due to the joint function of the proteins.

The detection of a *POLG2* variant might have implications for patient care: Individuals with mitochondriopathies are at an increased risk of complications related to volatile anesthetics, muscle relaxants, and some drugs such as valproic acid, statins, and metformin. Furthermore, certain antibiotics should be avoided or only be used with caution, as those substances might deteriorate mitochondrial disease [30]. However, whether these precautions also apply to *POLG2*-related disease has not yet been reported. Concerning the treatment, proven disease-modifying drugs are not available, and no data exist on whether mitochondrial enhancers, e.g., coenzyme Q10 or carnitine, help alleviate the symptoms.

Taken together, we provide evidence that the novel *POLG2* variant leads to impaired mitochondrial integrity suggesting its pathogenicity for the mitochondrial phenotype (ataxia and progressive external ophthalmoplegia) in the present family. Overall, the phenotypic spectrum in *POLG2*-related disease is broad and distinct in childhood- and adult-onset cases. Our work emphasizes the relevance of investigating *POLG2* as a nuclear gene in patients with cerebellar ataxia — as *POLG2* might currently not be included in all commercially available diagnostic ataxia panels — and further highlights the importance of the functional characterization of variants of uncertain significance to allow for meaningful genetic counseling.

### Supplementary Information

Below is the link to the electronic supplementary material.

Supplementary file1 (XLSX 25 KB)

Supplementary file2 Video 1: Representative excerpts of the neurological examination of the index patient, a 56-years old female, presenting with external ophthalmoplegia and severe cerebellar ataxia. (MOV 4921 KB) 

Supplementary file3 Video 2: The patient shows milder cerebellar ataxia compared to his sister, no external ophthalmoplegia, but dysmetria of saccades. (MOV 4839 KB) 

## Data Availability

Additional raw data supporting the conclusions of this article will be made available by the authors upon reasonable request.
